# Volatile Biomarkers in Breath Associated With Liver Cirrhosis — Comparisons of Pre- and Post-liver Transplant Breath Samples

**DOI:** 10.1016/j.ebiom.2015.07.027

**Published:** 2015-07-26

**Authors:** R. Fernández del Río, M.E. O'Hara, A. Holt, P. Pemberton, T. Shah, T. Whitehouse, C.A. Mayhew

**Affiliations:** a School of Physics and Astronomy, University of Birmingham, Birmingham B15 2TT, UK; b Department of Hepatology, University Hospital Birmingham NHS Trust, Birmingham B15 2TH, UK; c Critical Care and Anaesthesia, University Hospital Birmingham NHS Trust, Birmingham B15 2TH, UK

**Keywords:** AID, autoimmune liver disease, ALD, alcoholic liver disease, AUROC, area under receiver operator curve, CD, cryptogenic disease, GC, gas chromatography, HBV, hepatitis B virus, HCC, hepatocellular cancer, HCV, hepatitis C virus, ITU, intensive treatment unit, LQ, lower quartile, MS, mass spectrometry, OPU, out-patient clinic, PBC, primary biliary cirrhosis, PSC, primary sclerosing cholangitis, ppbv, parts per billion by volume, ppmv, parts per million by volume, PTR-MS, proton transfer reaction mass spectrometry, ROC, Receiver operating characteristics, TAC, transplant assessment clinic, TE, transient elastography, UKELD, United Kingdom model for end-stage liver disease, UQ, upper quartile, VOC, volatile organic compounds, VMR, volume mixing ratio, BMI, body mass index, Breath analysis, Cirrhosis, Diagnosis limonene, Liver transplant, PTR-MS, Volatile organic compounds

## Abstract

**Background:**

The burden of liver disease in the UK has risen dramatically and there is a need for improved diagnostics.

**Aims:**

To determine which breath volatiles are associated with the cirrhotic liver and hence diagnostically useful.

**Methods:**

A two-stage biomarker discovery procedure was used. Alveolar breath samples of 31 patients with cirrhosis and 30 healthy controls were mass spectrometrically analysed and compared (stage 1). 12 of these patients had their breath analysed after liver transplant (stage 2). Five patients were followed longitudinally as in-patients in the post-transplant period.

**Results:**

Seven volatiles were elevated in the breath of patients versus controls. Of these, five showed statistically significant decrease post-transplant: limonene, methanol, 2-pentanone, 2-butanone and carbon disulfide. On an individual basis limonene has the best diagnostic capability (the area under a receiver operating characteristic curve (AUROC) is 0.91), but this is improved by combining methanol, 2-pentanone and limonene (AUROC curve 0.95). Following transplant, limonene shows wash-out characteristics.

**Conclusions:**

Limonene, methanol and 2-pentanone are breath markers for a cirrhotic liver. This study raises the potential to investigate these volatiles as markers for early-stage liver disease. By monitoring the wash-out of limonene following transplant, graft liver function can be non-invasively assessed.

## Highlights

• Breath volatiles were compared for cirrhotic patients and controls and pre- and post-liver transplant.
• Three volatiles (limonene, methanol, 2-pentanone) have been found to have excellent diagnostic capabilities.
• Limonene shows washout characteristics following transplant supporting a hypothesis that it accumulates in fat.

There are numerous previous studies investigating breath volatiles in patients with liver disease but with conflicting results. It is impossible to tell which volatiles from previous studies may be false discoveries, and which are actually associated with the disease. We measured breath samples in patients and controls and in patients after transplant. Methanol, 2-pentanone and limonene show differences not only between patients and controls but also in cases pre- and post-transplant and have excellent diagnostic capabilities. We show evidence that limonene accumulates in the body, probably because the cirrhotic liver fails to metabolise dietary limonene.

## 1 Introduction

The publication of the 2014 Lancet Commission on liver disease has highlighted how the burden of liver disease in the UK has risen sharply over the past few decades and that it poses a major public health issue (Williams et al., 2014). It is the only major cause of mortality and morbidity which is on the increase in England, while at the same time decreasing in most other European countries, with cirrhosis accounting for 83% of deaths (Davies, 2012). It is the third biggest cause of premature mortality, with three quarters of liver deaths due to alcohol (Williams et al., 2014). Liver disease has a widespread effect not only to the patient, encompassing physical and psychological morbidity and mortality, but also incurring significant societal costs. One of the main difficulties is that often patients do not present symptoms or signs until the disease is advanced. Even then diagnosis is difficult and the symptoms and signs are often general and can be mistaken for other pathologies. Non-invasive diagnostic techniques currently used, namely serum biomarkers and transient elastography (TE) are not ideal. Serum biomarkers are not liver specific and TE results require an expert clinician for interpretation (Castera et al., 2015).

Among the ten key recommendations in a recent Lancet report is to strengthen the detection of early-stage liver disease, which is essential to reduce disease progression (Williams et al., 2014). Analysis of volatiles in the breath has the potential to deliver this, but only if chemical compounds can be found that are unambiguously associated with a diseased liver.

To date, the use of breath volatiles for medical diagnosis has met with limited success. Confounding factors, such as volatiles present in the environment, contamination in the sampling procedures and poor sampling methods, have meant that there is a great deal of uncertainty in volatile discovery (Kwak and Preti, 2011). Problems of bias and false discovery in biomarker discovery research have been widely reviewed (Broadhurst and Kell, 2006; Ransohoff, 2005).

Previous studies investigating breath volatiles in patients suffering with liver disease have proposed a large number of possible biomarkers (Millonig et al., 2010; Morisco et al., 2013; Sehnert et al., 2002; Solga et al., 2006; Tangerman et al., 1983; Van den Velde et al., 2008; Dadamio et al., 2012; Friedman et al., 1994; Hanouneh et al., 2014; Khalid et al., 2013, 2014; Shimamoto et al., 2000; Vollnberg et al., 2009; Verdam et al., 2013), but generally different studies report different volatiles. Various GC–MS studies found raised levels of many volatiles in the breath of patients with liver disease, including dimethyl sulfide, acetone, 2-butanone, 2-pentanone, β-pinene, α-pinene, and limonene (Van den Velde et al., 2008; Dadamio et al., 2012; Friedman et al., 1994; Khalid et al., 2013). Studies using soft chemical ionization mass spectrometric techniques have reported volatiles such as acetaldehyde, ethanol, isoprene, benzene, methanol, 2-butanone, 2- or 3-pentanone, heptadienol, and a monoterpene (limonene) (Morisco et al., 2013). Although the results of these studies are extremely encouraging, few volatile organic compounds (VOCs) are common to more than two or three studies and it is not useful to have hundreds of putative markers. Furthermore some volatiles, which have been proposed as biomarkers for liver disease, such as isoprene, acetone and ethanol, are not specific enough because they are possible biomarkers for other diseases or arise from numerous normal metabolic processes. If breath analysis is to progress to clinical utility, then markers must be definitively associated with the disease in question.

All previous studies can be regarded as hypothesis-generating, in that they do not follow up in a second group to confirm the putative biomarkers. We report here a two-stage breath biomarker discovery process: breath samples from a group of patients suffering from liver disease are first compared to breath samples from healthy controls; post-transplant breath samples are then compared with a sub-cohort of these patients who went on to have a liver transplant. A set of putative volatile markers is first determined by comparing patients with controls, and then pre- and post-transplant breath samples are examined to look for intra-individual differences in these volatiles. In this way, this study is hypothesis-led and uses patients as their own controls, thereby reducing the risk of false discovery. Furthermore, the use of patients' companions as controls and of room air samples minimizes the influence of any exogenous volatiles present in the home and hospital as confounding factors.

## 2 Methods

### 2.1 Patients, Controls and Hospital Room Air

Patients were recruited at the University Hospital Birmingham from either the transplant assessment clinic or in wards after being admitted with hepatic encephalopathy. 31 patients suffering from liver disease participated in the pre-transplant measurements (F/M 8/23, mean age 55 years, min–max 27–71 years). There were a number of etiologies and 11 patients had more than one condition: alcoholic liver disease (N = 13), hepatocellular cancer (N = 10), cryptogenic (N = 4), hepatitis C (N = 5), primary sclerosing cholangitis (N = 4), primary biliary cirrhosis (N = 2), autoimmune liver disease (N = 1), hepatitis B (N = 3), non-alcoholic steatohepatitis (N = 1), and non-alcoholic fatty liver disease (N = 1). Of these 31 patients, 12 went on to have a liver transplant (F/M 4/8, mean age 48 years, min–max 27–58 years). One additional patient (F, age 53 years) was recruited into the post-transplant study. Table 1 summarises the details of the 13 patients who had a liver transplant (12 from the pre-group), with patients being identified by sex (F or M) and a number. In addition to pre-transplant diagnostics, definitive diagnoses by histopathological examination of the explanted liver are provided. All but one (F2) were diagnosed with cirrhosis by standard liver function tests and biopsy. F2 was admitted suffering with hepatic encephalopathy, and histopathology of the explanted liver gave a diagnosis of severe hepatitis with multiacinar necrosis. Supplementary Table 1 shows demographic information for all patients including medications they were taking at the time of the pre-transplant sample.

For 28 pre-transplant measurements, breath samples from the patients' companions were taken. For the other three, two came alone to clinic and the other's companion declined to take part. Two additional controls were therefore recruited, one was a ward nurse and the other was a visitor to the hospital in ITU. These controls, while not related to the patient, had been in the same room for several hours prior to sampling so that confounding factors associated with volatiles present in the room environment were taken into consideration. In total 30 controls (F:M 23:7, mean age 44 years, min–max 20–75 years) took part in the study. The larger number of females in the control group arose due to the tendency of men coming to the clinic accompanied with their wives. While this means the control group is not ideally matched, there is no consistent evidence of dependences of volatile breath composition on sex (Kwak and Preti, 2011; Ellis and Mayhew, 2014). In confirmation of this we also found no correlation between sex and VOCs in either our control or patient groups. We consider that inhaled VOCs have a greater potential to confound biomarker discovery. As the majority of the companions were living with the patients, they provided an ideal control for exposure to exogenous volatiles in the home environment. VOCs inhaled at home, or in transit, may well still be present in breath for hours or days after inhalation and the biological half-life of inhaled VOCs is not well known (Beauchamp, 2011).

All study subjects were asked to complete a detailed questionnaire which included details on their home environment, diet, smoking status, health and medications. Participants were asked if they had consumed fruit and fruit juices and fruit flavoured drinks as a normal part of their diet, and, if so, to provide details on quantity and how long before the breath sampling these had been consumed.

Hospital room air was collected every time breath samples were taken so that any exogenous volatiles, such as isopropanol coming from hand gels resulting in product ions at *m/z* 43 and *m/z* 61, could be taken into consideration.

### 2.2 Breath Sampling Protocol

**Fig. 1:**
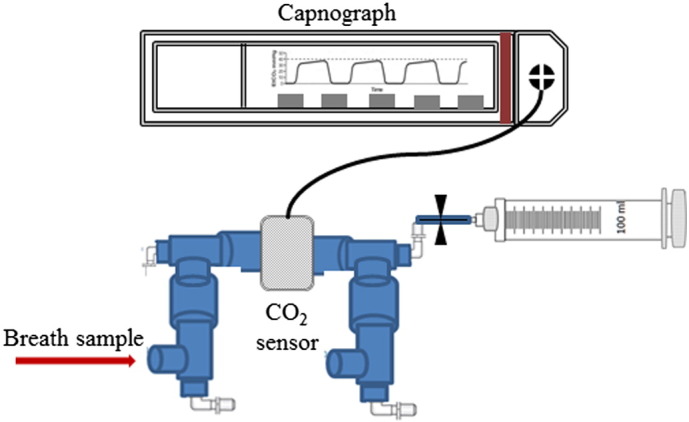
Schematic of the breath sampling device. Breath samples are only drawn into the glass syringe once the capnograph shows that the alveolar phase of the exhaled breath has been reached. Typically 3–4 breaths are needed to fill a syringe to 100 ml.

There is no agreed standard for the collection of breath for volatile analysis and uncontrolled breath sampling has been shown to be unreliable (Schubert et al., 2001; O'Hara et al., 2008). Therefore, capnography controlled sampling was used to collect only the alveolar phase of the breath. Subjects were in a relaxed state throughout the measurements and were either in a seated or lying position. They were asked to breathe normally into a gas tight respiratory system (Intersurgical Limited) containing an in-line CO_2_ mainstream sensor connected to a fast-time response capnometer (Capnogard 1265 Novametrix Medical Systems Inc.). A 100 ml glass syringe (Sigma-Aldrich) was coupled to the tubing using a 3-way luer-lock stopcock (Braun Medical Limited). When the alveolar plateau on the capnograph was observed, a breath sample was manually drawn from the subject's breath stream into the syringe. Three to four breaths samples were collected for each 100 ml syringe, and four replicates of these were taken for each subject. Glass syringes were used, because our tests showed that they have no contaminating volatiles. Fig. 1 schematically shows the sampling system used.

After collection, the syringes were sealed using the luer lock fitting. They were transported from hospital to laboratory (a 10 minute outdoor walk) in an opaque storage box. Once at the laboratory, the syringes were placed inside an incubator set at 40 °C.

All samples were mass spectrometrically analysed within 2 h of collection. For the measurements, syringes were taken out of the incubator and immediately placed into a purpose designed heating bag (Infroheat, Wolverhampton) maintained at a constant temperature of 40 °C in order to limit condensation, which could otherwise lead to volatile loss (Beauchamp et al., 2008). The luer stopcock was coupled to a Swagelok fitting and connected directly to the inlet of the analytical device, a proton transfer reaction mass spectrometer (PTR-MS). The inlet flow was set at 10–15 ml/min and the drift tube and inlet lines were maintained at 45 °C. The syringes are gas tight and have minimal friction such that atmospheric pressure is sufficient to push the plunger in smoothly so that the breath sample is being drawn into the instrument at a constant flow.

### 2.3 Analytical Measurements

PTR-MS is a platform technology designed to detect low concentrations of volatiles (less than parts per billion by volume). Hence it has found use in many analytical applications ranging from drug detection through to industrial pollution (Ellis and Mayhew, 2014; Lindinger et al., 1998; Agarwal et al., 2011; de Gouw et al., 2011; Jurschik et al., 2012). Details of the instrument used, a PTR-Quad-MS (IONICON Analytik GmbH), and how it operates are described in detail in the literature (Ellis and Mayhew, 2014; O'Hara et al., 2008; Lindinger et al., 1998). In brief, it exploits the reactions of protonated water with neutral volatiles (M), usually leading to a protonated parent (MH^+^). If dissociative proton transfer occurs then it is not extensive in terms of the number of resulting product ions. Operational parameters used for this investigation were those previously reported (O'Hara et al., 2009a,b). Namely, the drift-tube was maintained at a pressure of 2.07 ± 0.01 mbar and temperature of 45 ± 1 °C. The voltage across the drift-tube was set at 600 V, which is sufficiently high to reduce water clustering to reagent and product ions by collision induced dissociation.

A *m/z* range of 20 to 200 amu was scanned with a dwell time of 0.5 s per atomic mass unit. Mass spectra of the breath samples were recorded from the average of three cycles for each of the four syringes, for every participant. These four spectra were averaged to provide one data set for each subject with the uncertainty expressed as the standard error of the mean for the four syringes.

The intensities of the product ion(s) associated with a given volatile were converted to volume mixing ratios (VMR) in units of nmol/mol by use of a standard procedure that relies on a calculated, compound-specific, collisional reaction rate coefficient, determined using the effective translational temperature of the reagent ions (Ellis and Mayhew, 2014).

To help identify product ions, pure samples of key volatiles were individually measured using PTR-MS to establish the *m/z* values of the product ions.

### 2.4 Data Analysis and Statistics

Room air contamination is a potential confounding factor in breath analysis and has been the subject of much discussion. If the intensity of a product ion has a significant contribution coming from room air, then care must be taken when using it as a biomarker. There is no simple correction which can be applied to account for inhaled volatile concentrations, and it has been shown that simply subtracting the room air concentration is too simplistic (Schubert et al., 2005; Spanel et al., 2013; Pleil et al., 2013). For this study, a filter was applied such that only ion signal intensities in the breath sample that were at least twice that in the room air samples in at least half of the patients were retained for analysis. This resulted in a set of 40 product ions for analysis. (The m/z values and normalised counts per second are provided in Supplementary Table 2, many of which are well known including m/z 33 (methanol), m/z 45 (acetaldehyde), m/z 47 (ethanol), m/z 59 (acetone) and m/z 69 (isoprene).

The data sets for each volatile of interest were assessed using a Shapiro–Wilks test and were found not to be normally distributed so non-parametric tests were used. IBM SPSS version 22 was used for all statistical analysis. Mann–Whitney U-tests determined which *m/z* values differed between the patients and controls. A Wilcoxon signed rank test was used to determine which volatile concentrations differed between pre- and post-transplant breath samples. To compare blood chemistry values with breath volatiles Kendall's tau-b correlation coefficients were measured. Receiver Operating Characteristic (ROC) curves were used to determine the diagnostic accuracy of volatiles.

## 3 Results

**Fig. 2:**
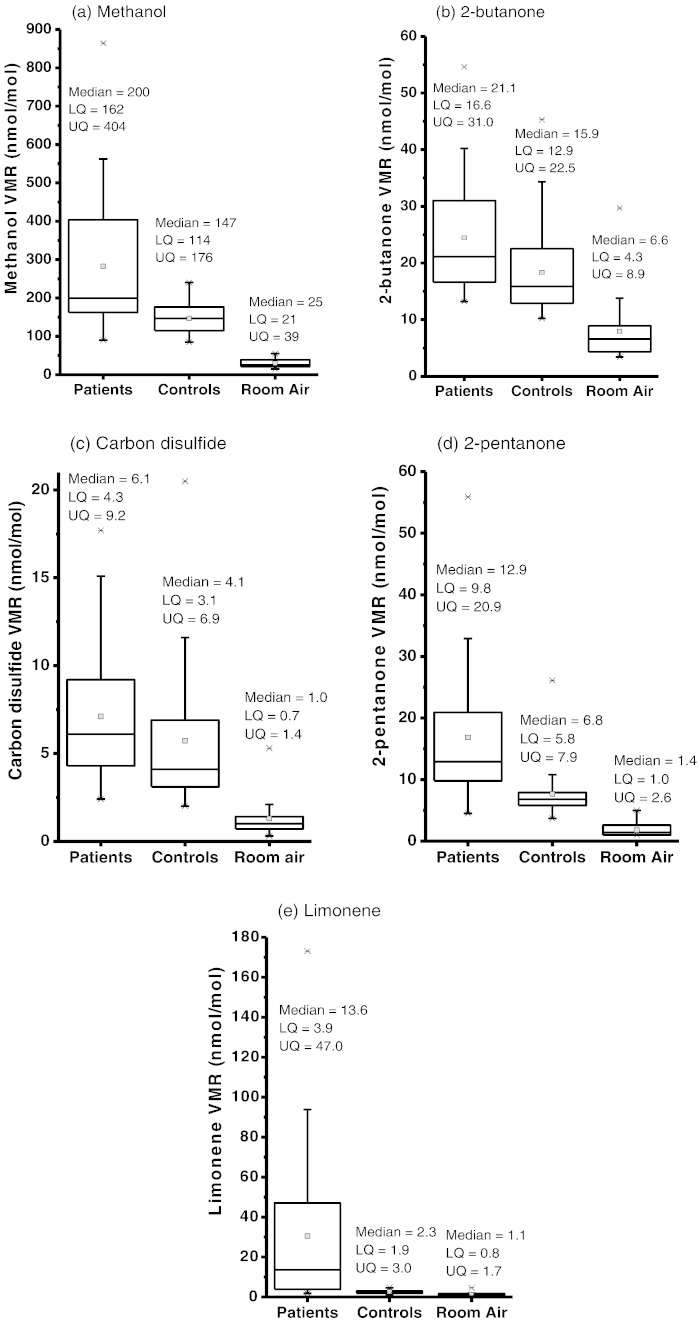
Boxplots showing in units of nmol/mol lower quartile (LQ), median, mean and upper quartile (UQ) calculated volume mixing ratios (VMRs) for (a) methanol, (b) 2-butanone, (c) carbon disulfide, (d) 2-pentanone, and (e) limonene for 31 patients with liver cirrhosis, 30 controls and room air samples. Whiskers are 1.5 times the inter-quartile range and outliers are depicted by a star.

For the first stage of our study, a Mann–Whitney U-test with a significance level of 95% was used to compare the 40 product ion signal intensities used in our analysis between patients and controls. Of these, eight showed significant differences in intensities between patients and controls. Their *m/z* values and significance (p value in brackets) are 33 (< 0.001), 73 (0.004), 77 (0.035), 81 (< 0.001), 87 (< 0.001), 89 (0.03), 135 (0.019) and 137 (< 0.001). In the second phase, pre- and post-transplant intensities of these eight ions were compared using a Wilcoxon Signed Rank Test for paired samples with a significance of 95%. This eliminated *m/z* 89 and 135 from the putative marker set. Of the remaining ions, *m/z* 33 is assigned to be protonated methanol (CH_3_OH_2_^+^) (Lindinger et al., 1997). Based on previous GC and GC–MS studies (Sehnert et al., 2002; Van den Velde et al., 2008; Dadamio et al., 2012), we tentatively identify *m/z* 73 as protonated 2-butanone (C_4_H_8_OH^+^), *m/z* 77 as protonated carbon disulfide (CS_2_H^+^), *m/z* 87 as protonated 2-pentanone (C_5_H_10_OH^+^), and *m/z* 81 and 137 as limonene. (*m/z* 81 is a fragment ion (C_6_H_9_^+^) resulting from dissociative proton transfer and *m/z* 137 is protonated limonene (C_10_H_17_^+^).) VMRs of these volatiles in room air, patient and control samples are shown in Fig. 2 for (a) methanol, (b) carbon disulfide, (c) 2-butanone, (d) 2-pentanone, and (e) limonene (sum of the intensities of the *m/z* 81 and *m/z* 137 product ions). The median, mean, lower quartile (LQ), and upper quartile (UQ) of the VMRs in units of nmol/mol for each volatile are shown. It is clear from Fig. 2 that the presence of the volatiles in room air has a negligible effect on the concentrations in the breath samples. Furthermore, the analysis is based on comparisons and the patients and controls should be affected similarly by the room air contaminations.

The pre-transplant and post-transplant VMRs for methanol, carbon disulfide, 2-butanone, 2-pentanone and limonene for all of our participants who underwent liver transplants (4 females (F1–F4) and 8 males (M1–M8)) are provided in Table 2. It should be noted that the number of days between collecting the pre-transplant and post-transplant breath samples is variable, because it is not possible to control when subjects are available or when a donor liver would be found. Only for one of the patients, F2, were we able to collect a pre-transplant breath sample just prior to surgery. However, and independent of when the pre- and post-transplant breath samples were taken, the results in Table 2 clearly demonstrate that the pre-transplant concentrations of these volatiles are, for the majority of patients, higher than the post-transplant levels for most patients. Limonene shows the largest average decrease and also decreased in all patients post-transplant. Post-transplant concentrations of limonene dropped to within the normal control range (median (LQ, UQ) being 2.3 nmol/mol (1.9, 3.0)) within a number of days for all but one of the patients, M4, for whom limonene was found to be high even some months after transplant.

**Supplementary Fig. 1:**
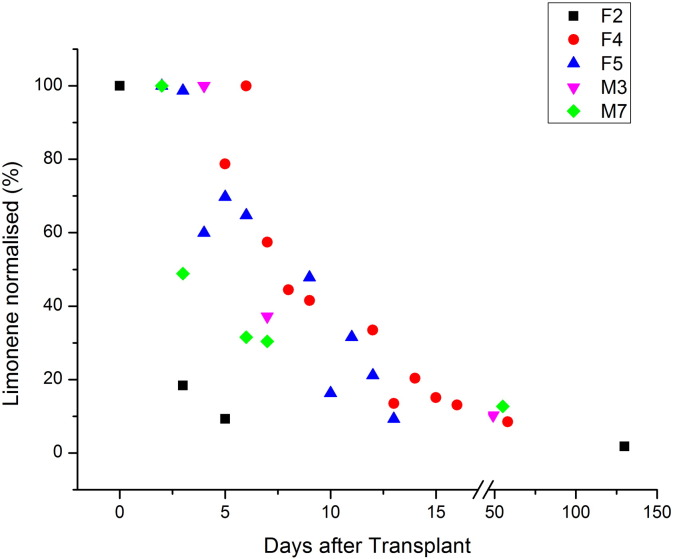

**Fig. 3:**
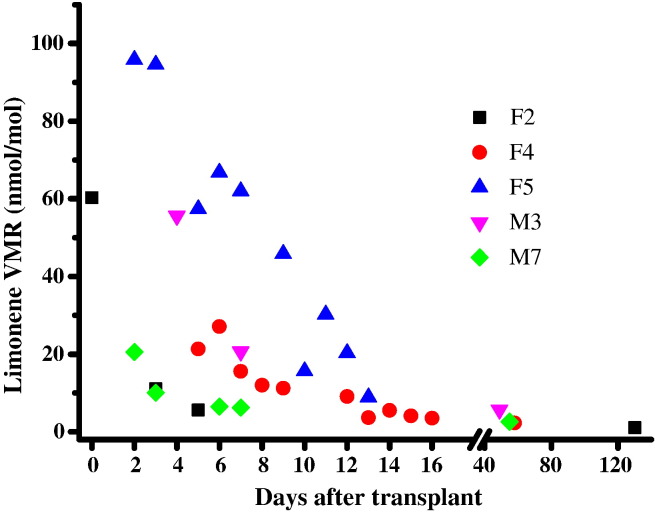
Longitudinal changes in volume mixing ratios (VMRs) in nmol/mol for limonene at given days after liver transplant for patients F2, F4, F5, M3, and M7. The data point at day 0 for F2 was taken just before transplant surgery.

In order to gain an insight on how the methanol, carbon disulfide, 2-butanone, 2-pentanone and limonene breath VMRs changed over a period of time after transplant, five patients (F2, F4, F5, M3 and M7) participated in a longitudinal study. The key result is that limonene VMRs dropped gradually following transplant surgery, as illustrated in Fig. 3. (The same data for limonene presented as normalised to the highest intra-individual value are shown in Supplementary Fig. 1.) This concentration time dependence was not observed for methanol, carbon disulfide, 2-butanone and 2-pentanone. Their VMRs were found to have dropped to within the normal range by the time of the first post-transplant measurement.

**Fig. 4:**
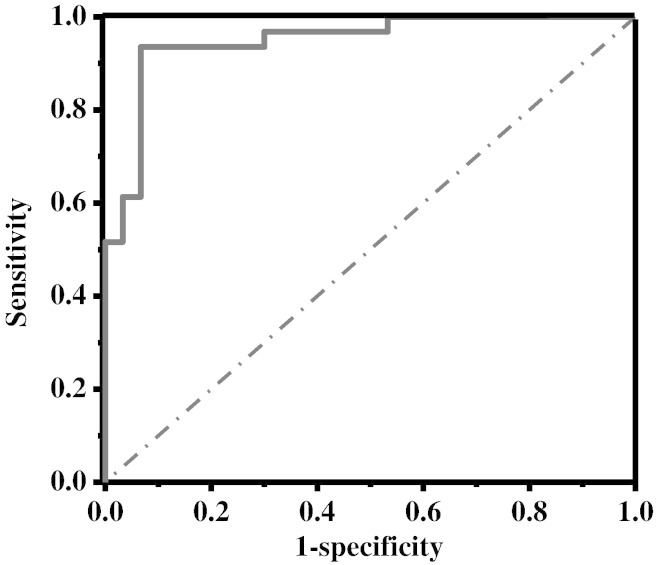
Receiver operating characteristic curve for a combination of methanol, 2-pentanone and limonene data in the study groups.

Taken together, the box plots (Fig. 2), the ratio of the pre- and post-transplant VMR values and the significance values given above imply that ions at *m/z* 33, 81, 87 and 137 are the ones that are most diagnostically useful. This is confirmed by ROC curve analyses. Individually, limonene is found to provide the most predictive power (AUROC — 0.91 (standard error 0.04)). However, the best accuracy is achieved by combining the data from methanol, 2-pentanone and limonene. The VMRs for limonene, methanol and 2-pentanone were normalised to the highest patient value for that volatile. These normalised fractions were simply added with no weightings. Fig. 4 shows a ROC curve for the combined data. The AUROC is 0.95 (standard error 0.03) and achieves a sensitivity of 97% with a specificity of 70%.

Clinical chemistry data for the patients for whom blood data were available were analysed for possible correlations with limonene, methanol and 2-pentanone. Correlations were checked for alanine aminotransferase, alkaline phosphatase, aspartate transferase, albumin, total bilirubin, creatinine, neutrophils, platelets, potassium, prothrombin/international normalised ratio, and the United Kingdom Model for End-Stage Liver Disease (UKELD). Kendall's tau-b analysis showed only one correlation with a significance score below 0.05. This was for methanol with UKELD which had a Kendall's tau-b coefficient of 0.237 (significance 0.042). Over 33 correlations were tested and no multiple testing correction was applied so it is possible that this is a coincidental finding.

Volatile concentrations were examined for correlations with disease etiology. Owing to the small sample size and large number of etiologies, this was only feasible for the 13 patients with ALD versus the other 18 patients. Limonene was higher (p = 0.020) in the ALD group than the rest, with median (LQ, UQ) of 19.7 nmol/mol (9.2, 63.9) for ALD versus 6.1 nmol/mol (2.9, 16.6) for all other etiologies. Methanol, 2-pentanone, 2-butanone and carbon disulfide showed no statistically significant difference.

Correlations between the 7 volatiles of interest in the putative marker set were examined both within the patient and the control group using a Kendall's tau-b test. In the patient group, there were 8 correlations with a significance score of < 0.05. Results for all 21 correlations are shown in Supplementary Table 3. In the control group, only two were significant, limonene with m/z 135 (p = 0.016) and 2-butanone with carbon disulfide (p < 0.001). These were also found in the patient group. Limonene correlated significantly with 2-butanone (p = 0.004), carbon disulfide (p = 0.034), m/z 89 (p = 0.001) and m/z 135 (p < 0.001) but not with methanol or 2-pentanone. This suggests that the mechanisms for the presence of limonene and that of methanol and 2-pentanone are independent. 21 correlations were examined with no multiple-testing correction applied, therefore some correlations may be coincidental.

Correlations between the concentrations of volatiles and demographic markers such as age, BMI and sex were also checked. No significant correlations were found.

## 4 Discussion

A major aim of this study is to determine the viability of breath analysis as a non-invasive technique for monitoring/diagnosing liver disease by identifying volatiles in the breath which are a consequence of the disease. In this investigation, the monitoring of volatiles in breath following a dramatic change in the condition of the patient, namely a liver transplant, has provided a method to attribute three diagnostically useful volatiles to the cirrhotic organ itself. These are methanol, 2-pentanone, and limonene, with that of limonene being the most significant.

Limonene has been found in previous breath volatile studies to be elevated in the breath of patients with cirrhosis compared with controls (Morisco et al., 2013; Dadamio et al., 2012; Friedman et al., 1994). It has also been observed in the breath of healthy volunteers; limonene levels found in our control group are comparable to those previously observed in healthy human volunteers (Mochalski et al., 2013). Limonene is not produced in the human body. It is a common compound naturally found in many foods and drinks; hence it would be difficult to avoid ingesting. Within the control and patient groups, we found no association between breath limonene and diet and no correlation between having a self-reported large amount of fruit consumption and breath limonene concentrations. Once in the blood stream, limonene is metabolised by the P450 enzymes CYP2C9 and CYP2C19 to the metabolites perillyl alcohol, trans-carveol and trans-isopiperitenol (Miyazawa et al., 2002). It has been found that levels of the enzyme CYP2C19 are reduced in patients with cirrhosis and that levels inversely correlate with severity of cirrhosis. Moreover, of four P450 enzymes tested in patients with liver disease, metabolism by CYP2C19 was found to decrease at the earliest stage of disease (Frye et al., 2006). This is suggestive that the observed raised concentrations of limonene in breath arise from the inability of a cirrhotic liver to produce the appropriate metabolic enzyme (Morisco et al., 2013). Patient M4 is anomalous in this respect, as his breath limonene concentrations do not drop to the normal range post-transplant. Although his graft liver function blood tests were found to be normal, our results suggest that this patient's new liver is not producing sufficient enzyme to fully metabolise limonene.

Owing to its lipophilic properties, we propose that limonene which is not metabolised by the liver accumulates in the fat of patients suffering from liver disease. Limonene has a blood/air partition coefficient of 36 and an olive oil/blood partition coefficient of 140 (Falk et al., 1990). Assuming that the olive oil/blood partition coefficient is close to a body fat/blood partition coefficient, a breath concentration of 1 part per billion by volume (ppbv) would translate to a fat concentration of approximately 5 parts per million by volume (ppmv). Our highest recorded breath VMR is 170 nmol/mol which implies a concentration in fat of the order of 850 ppmv. A study involving women with early-stage breast cancer taking a high oral dose of limonene (2 g/daily for 2–6 weeks before surgery) found that mean limonene concentration in breast tissue was 41.3 ± 49.9 μg/g which is much higher than that found in a control group (0.08 ± 0.13 μg/g) (Miller et al., 2013). Breast tissue is primarily composed of fat (Boston et al., 2005). This supports our hypothesis that unmetabolised limonene accumulates in fat tissue. Following transplant, the metabolism of limonene increases, but it takes time for the limonene to be released from the fat into the blood stream. This, we propose, explains the observed time dependence on limonene VMRs in the breath after transplant. A similar wash-out behaviour is not observed for methanol and 2-pentanone presumably owing to their low solubilities in fat (Griffin et al., 1999; Sangster, 1989).

Some medications are known to be CYP2C9 and CYP2C19 substrates and inhibitors. We therefore looked into the possibility that medications could affect limonene concentrations. Twenty patients were taking a CYP2C19 substrate (lansoprazole, omeprazole, propanalol, esomeprazole), 2 were taking a CYP2C9 substrate (naproxen, carvedilol), 6 were taking both a CYP2C9 and CYP2C19 substrate and 1 was taking a CYP2C9 inhibitor (sulfamethaoxazole). Our results show no associations of any medications which are CYP2C9 and CYP2C19 substrates or inhibitors with VMRs of breath limonene. Moreover, nine patients who were taking enzyme substrates before transplant were still taking them after transplant.

Of interest is the correlation between limonene and *m/z* 135, because this product ion may come from perillyl alcohol (C_10_H_16_O), a metabolite of limonene. Studies by us (unpublished) have shown that the reaction of H_3_O^+^ with perillyl alcohol leads to a dominant product ion C_10_H_15_^+^ resulting from dehydration of the protonated parent. Dehydration following protonation is a common reaction process observed with many alcohols (Brown et al., 2010). Morisco et al.(2013) also noted an ion at *m/z* 135 from patients with cirrhosis, but they assigned this to a terpene related compound. The fact that a correlation of *m/z* 135 and limonene (p < 0.001) is also significant in the control group lends support to our assignment, because one would expect levels of a compound and its metabolite to be correlated in a group with well-functioning livers. It is also of interest to note that the correlation between limonene and *m/z* 89 has a very low p-value, but that *m/z* 89 shows no discrimination between pre- and post-liver transplant. This is suggestive that *m/z* 89 arises from an independent process related to the patient's illness, but is not related to the cirrhosis itself.

The enhanced levels of methanol and 2-pentanone in pre-transplant patients could come from a number of sources, including diet. Elevated levels of methanol have been reported following consumption of alcohol or large quantities of fruit (Lindinger et al., 1997). It is a product of the degradation of pectin by colonic bacteria (Siragusa et al., 1988), and of metabolism of the sweetener aspartame (Stegink et al., 1989). However, and in agreement with Morisco et al. (2013) we find that fruit consumption cannot explain the increased methanol concentrations in the breath of liver patients compared to controls. Alcoholic drinks are a source of methanol, but only one patient reported that he had drunk alcohol within the 24 h prior to the breath sampling. Methanol is metabolised in humans in the liver, mainly by alcohol dehydrogenase (Skrzydlewska, 2003), so it is possible that this mechanism is impaired when a liver becomes cirrhotic. Morisco et al.(2013), also found elevated methanol in cirrhotic patients versus healthy controls. The source of 2-pentanone in breath is unknown (King et al., 2010). It has been found in human breath, faeces, skin and urine (de Lacy et al., 2014), and it has been suggested that lung cells produce 2-pentanone (Filipiak et al., 2010). 2-pentanone was suggested as a biomarker for liver disease by three previous studies (Morisco et al., 2013; Van den Velde et al., 2008; Vollnberg et al., 2009).

In conclusion, we have performed a two-stage study which compares volatiles in the breath of pre-transplant cirrhotic patients with controls followed by pre- and post-transplant breath samples. This has resulted in an assignment of methanol, 2-pentanone and limonene as markers in exhaled breath for the cirrhotic liver. We have demonstrated that limonene can also be used for assessing liver function following transplant by monitoring wash-out. Our study links limonene with the diseased organ itself, rather than simply the diseased patient as a whole. Breath volatiles have the advantage of offering non-invasive testing, but also offer the opportunity to assess the global function of the liver, rather than a localised test such as a biopsy. Our study raises the possibility of a pharmacokinetic-based test for assessing liver function which could be used for diagnosing liver disease, i.e. where a known quantity of limonene is administered and its wash-out in breath is assessed over time. Importantly, this study provides a set of biomarkers which can be used in future studies to assess the potential of breath analysis for the diagnosis of early-stage liver disease.

The following are the supplementary data related to this article.

Supplementary Table 1 Demographic information for the patient group. Medications are those which they were taking at the time of the pre-transplant breath sample, except for F5, who was only sampled post-transplant. BMI = body mass index. The weight used for the BMI calculation is the one given in the patient's notes closest to the date for the pre-transplant sample. AID = autoimmune liver disease, ALD = alcoholic liver disease, CD = cryptogenic disease, HBV = hepatitis B virus, HCC = hepatocellular cancer, HCV = hepatitis C virus, LF = liver failure, NAFLD = non-alcoholic fatty liver disease, NASH = non-alcoholic steatohepatitis, PBC = primary biliary cirrhosis, PSC = primary sclerosing cholangitis.

Supplementary Table 2 Normalised counts per second (using area under the peak) (NCPS) for the 40 measured ion peaks which were included in the statistical analysis for patients, controls and room air. Median (lower quartile LQ, upper quartile UQ) and range (minimum–maximum) are provided. NCPS refers to normalising to 50 million counts per second for the sum of the reagent ions m/z 19 (hydronium) and m/z 37 (the protonated water dimer). Note the number of room air samples are less than those for patients and controls because some patients were recruited in the same clinic at the same time.

Supplementary Table 3 Correlations between volatiles for the 31 patients and 30 controls in the putative marker set. Significance scores below 0.05 are highlighted in bold.

## Conflicts of Interest

All authors have declared no conflicts of interest.

## Contributions

MEOH and CAM in discussion with AH and TS were responsible for the conception of this study. RFdR was responsible for the design and development of the breath sampling system, taking the majority of the breath samples, for proposing the longitudinal study, and for undertaking most of the mass spectrometric measurements. RFdR and MEOH were responsible for the data analysis of the mass spectrometric files. MEOH was responsible for obtaining ethical approval, patient recruitment, study management, statistical analyses of the data and assisted with some of the sample collection and measurement. CAM, MEOH and RFdR wrote the paper with input from PP, AH, TS, and TW. PP, AH, TS, and TW were responsible for the provision of patients and for providing the medical context. The final paper has been approved by all authors.

## Ethics Approval

The regional ethics committee of Camden and Islington, London approved this study (REC reference: 13/LO/0952). Informed consent was obtained from each volunteer.

## Funding Sources

This work was in part funded through the Proton Ionisation Molecular Mass Spectrometry (PIMMS) Initial Training Network which is supported by the European Commission 7th Framework Programme under Grant Agreement Number 287382. MEOH thanks the Daphne Jackson Trust for a fellowship and the Engineering and Physical Sciences Research Council and University of Birmingham for her sponsorship. We thank the Wellcome Trust (grant number 097825/Z/11/B) for an Institutional Strategic Support Fund. We acknowledge the support of the National Institute of Health Research Clinical Research Network (NIHR CRN).

## Figures and Tables

**Table 1:** Liver transplant patient details, including sex (female F, male M), age, initial diagnosis, histopathological results, location of pre-transplant and post-transplant breath sampling, and the number of days prior to and after transplant when breath samples were collected. Diseases include autoimmune liver disease (AID), alcoholic liver disease (ALD), cryptogenic disease (CD), hepatitis B (HBV), hepatitis C (HCV), hepatocellular cancer (HCC), primary biliary cirrhosis (PBC) and primary sclerosing cholangitis (PSC). Breath samples were taken at various locations including the intensive treatment unit (ITU), out-patient clinic (OPC), transplant assessment clinic (TAC), and in wards.

Patient ID	Age (yr)	Initial diagnosis	Histopathological results	Location of pre-transplant breath sample	Pre-transplant breath sample: days before transplant	Location of post-transplant breath sample	Post-transplant breath samples: days after transplant
F1	27	AID	Severe steatohepatitis (PSC)	TAC	54	OPC	65
F2	49	Liver Failure	Severe hepatitis with multiacinar necrosis, seronegative hepatitis	ITU	0^1^	OPC	3, 5, 130
F3	53	PBC	Cirrhosis (PBC)	TAC	74	OPC	45
F4	58	PSC	Cirrhosis (PSC)	TAC	83	Ward	5–8, 11–15, 18, 58
F5	53	ALD	Hepatocellular carcinoma, liver cirrhosis.(ALD, HCV)	–	–	Ward	2–6, 9–12
M1	54	ALD	Severe steatohepatitis	Ward	47	OPC	33
M2	45	ALD	Cirrhosis (ALD)	TAC	97	OPC	22
M3	53	ALD	Cirrhosis (ALD/HCV) Hepatocellular carcinoma (grade2)	TAC	179	Ward	4, 7, 48
M4	53	ALD, HBV, HCV	Cirrhosis (ALD, HCV, HBV)	TAC	21	OPC	126
M5	56	ALD, HCV, HCC	Cirrhosis (ALD, HCV, HCC)	TAC	125	OPC	61
M6	53	CD	Cirrhosis with mild steatohepatitis (aetiology possibly NASH but uncertain)	TAC	154	OPC	22
M7	36	CD	Cirrhosis of uncertain aetiology	TAC	180	Ward	2, 3, 6–8, 55
M8	67	ALD	Cirrhosis (ALD)	ITU	14	Ward	6

The pre-transplant breath sample for patient F2 was taken approximately 10 min before the patient went into surgery. This patient was admitted with liver failure and hepatic encephalopathy.

**Table 2:** Calculated mean volume mixing ratios (VMRs) for methanol, 2-butanone, carbon disulfide, 2-pentanone, and limonene, in units of nmol/mol for pre- and post-transplant breath samples. The post-transplant values correspond to those for the last post-transplant sample given in Table 1. Measurement uncertainties are provided in brackets. The ratios of pre- to post-transplant concentrations are also provided.

Patient	Mean methanol VMRs nmol/mol			Mean 2-butanone VMRs nmol/mol			Mean carbon disulfide VMRs nmol/mol			Mean 2-pentanone VMRs nmol/mol			Mean limonene VMRs nmol/mol		
Pre	Post	Pre/post	Pre	Post	Pre/post	Pre	Post	Pre/post	Pre	Post	Pre/post	Pre	Post	Pre/post
F1	200 (6)	190 (3)	1.1 (0.04)	18 (1.2)	11 (0.2)	1.6 (0.1)	4.7 (0.4)	2.0 (0.4)	2.4 (0.5)	9.0 (0.8)	5.3 (0.6)	1.7 (0.2)	7.5 (0.6)	3.5 (0.6)	2.1 (0.4)
F2	90 (9)	230 (6)	0.4 (0.04)	16 (1.1)	15 (1.9)	1.1 (0.2)	2.4 (0.5)	20 (2.9)	0.1 (0.03)	8.3 (0.8)	7.3 (0.6)	1.1 (0.1)	60 (4.7)	1.1 (0.1)	54 (6)
F3	530 (2)	78 (5)	6.8 (0.4)	21 (1.7)	12 (2.6)	1.8 (0.4)	11 (1.3)	2.3 (0.3)	4.8 (0.8)	10 (1.2)	5.7 (0.3)	1.8 (0.2)	14 (0.7)	2.3 (0.2)	6.1 (0.6)
F4	560 (18)	71 (3)	7.9 (0.4)	19 (1.2)	12 (0.4)	1.6 (0.1)	4.7 (0.2)	2.3 (0.4)	2.0 (0.4)	25 (3.3)	6.1 (0.3)	4.1 (0.6)	11 (0.5)	2.3 (0.1)	4.8 (0.3)
M1	170 (2)	120 (15)	1.4 (0.2)	26 (1.4)	14 (1.4)	1.9 (0.2)	9.3 (0.6)	2.1 (0.4)	4.4 (0.9)	10 (0.5)	17 (2)	0.6 (0.1)	32 (0.7)	3.3 (0.5)	9.7 (1.5)
M2	430 (36)	81 (1)	5.3 (0.4)	13 (2.2)	15 (1.7)	0.9 (0.2)	3.1 (0.1)	2.4 (0.5)	1.3 (0.3)	23 (0.6)	6.2 (1.5)	3.7 (0.9)	94 (3.4)	1.6 (0.2)	59 (8)
M3	320 (32)	86 (7)	3.7 (0.5)	55 (3.1)	46 (4.2)	1.2 (0.1)	18 (2.0)	1.8 (0.2)	10 (1.6)	29 (1.0)	13 (1.2)	2.2 (0.2)	170 (1)	5.7 (0.2)	30 (1)
M4	490 (37)	93 (6)	5.3 (0.5)	38 (5.9)	23 (7.5)	1.7 (0.6)	8.5 (1.0)	2.6 (0.5)	3.3 (0.7)	38 (4.3)	9.9 (0.9)	3.8 (0.6)	110 (6)	55 (4)	2.0 (0.2)
M5	190 (3)	320 (1)	0.6 (0.01)	15 (0.5)	17 (1.3)	0.9 (0.1)	4.0 (0.4)	3.6 (0.1)	1.1 (0.1)	21 (1.3)	6.7 (0.5)	3.1 (0.3)	9.2 (0.5)	4.1 (0.2)	2.2 (0.2)
M6	230 (12)	79 (5)	2.9 (0.2)	30 (2.2)	12 (3.3)	2.5 (0.7)	13 (1.9)	2.1 (0.4)	6.2 (1.5)	13 (1.3)	5.1 (0.2)	2.5 (0.3)	120 (8)	4.8 (0.3)	25 (2)
M7	510 (21)	160 (8)	3.2 (0.2)	19 (0.7)	24 (4.2)	0.8 (0.1)	4.9 (0.9)	4.5 (0.7)	1.1 (0.3)	14 (2.6)	7.5 (0.3)	1.9 (0.4)	47 (2)	2.6 (0.1)	18 (1)
M8	180 (5)	86 (2)	2.1 (0.07)	21 (1.0)	12 (0.4)	1.8 (0.1)	6.8 (0.6)	1.9 (0.3)	3.6 (0.7)	8.8 (0.3)	13 (1.6)	0.7 (0.1)	7.7 (0.6)	3 (0.2)	2.6 (0.3)
