# Supplementary material for: Volatile Biomarkers in Breath Associated With Liver Cirrhosis — Comparisons of Pre- and Post-liver Transplant Breath Samples
Source: eBioMedicine. 2015 Jul 26;2(9):1243–50. doi: 10.1016/j.ebiom.2015.07.027 (PMC4588000; doi:10.1016/j.ebiom.2015.07.027)
Supplement: Supplementary Table 1 — Demographic information for the patient group. Medications are those which they were taking at the time of the pre-transplant breath sample, except for F5, who was only sampled post-transplant. BMI = body mass index. The weight used for the BMI calculation is the one given in the patient's notes closest to the date for the pre-transplant sample. AID = autoimmune liver disease, ALD = alcoholic liver disease, CD = cryptogenic disease, HBV = hepatitis B virus, HCC = hepatocellular cancer, HCV = hepatitis C virus, LF = liver failure, NAFLD = non-alcoholic fatty liver disease, NASH = non-alcoholic steatohepatitis, PBC = primary biliary cirrhosis, PSC = primary sclerosing cholangitis. [file mmc1.docx]

**Supplementary table 1.** Demographic information for the patient group. Medications are those which they were taking at the time of the pre-transplant breath sample, except for F5, who was only sampled post-transplant. BMI = body mass index. The weight used for the BMI calculation is the one given in the patient’s notes closest to the date for the pre-transplant sample. AID = Autoimmune liver disease, ALD = Alcoholic Liver Disease, CD = Cryptogenic disease, HBV = Hepatitis B virus, HCC = Hepatocellular cancer, HCV = Hepatitis C virus, LF = liver failure, NAFLD = Non Alcoholic Fatty Liver Disease, NASH = non-alcoholic steatohepatitis, PBC = Primary biliary cirrhosis, PSC = Primary sclerosing cholangitis.

| **Study ID** | **Sex** | **Age** | **BMI** | **diagnosis** | **Smoker** | **Medications** |
| --- | --- | --- | --- | --- | --- | --- |
| F1 | F | 27 | 25.8 | AID | yes | prednisolone, tacrolimus (prograf), azathioprine, lansoprazole, ursodeoxycholic acid, sulfasalazine |
| F2 | F | 49 | 31.2 | LF | no | esomeprazole before and after transplant, hydrocortisone, flucanozole, metaclopramide, mycophenalate |
| F3 | F | 53 | 26.4 | PBC | no | ursodeoxycholic acid, paracetamol, ranitidine hydrochloride, vitamin B, thiamine, lactulose. |
| F4 | F | 58 | 32.4 | PSC | no | spironolactone, omeprazole, ciprofloxacin, thiamine, calcium carbonate |
| F5 | F | 71 | 28.0 | CD | no | carvedilol, lactulose, omeprazole |
| F6 | F | 52 | 21.5 | PBC | no | deoxycholic acid |
| F7 | F | 65 | 28.0 | NASH, HCC | no | carvedilol, omeprazole, rifaximin, spironolactone, gabapentin, fruesamide, novomix 30 (insulin) |
| F8 | F | 54 | 22.1 | HCV, HCC | yes | clamiparin, omeprazole |
| M1 | M | 54 | 33.7 | ALD | yes | lansoprazole |
| M2 | m | 45 | 21.1 | ALD | yes | Lactulose, Lansoprazole, Mirtazapine, paracetamol, Propanolol, Adcal D-3, ciprofloxacin, Furosemide, spironolactone |
| M3 | M | 53 | 32.5 | ALD | no | Thiamine, Omeprazole, bisodol, vitamin B, amitryptiline, lactulose, topical steroid for psoriasis |
| M4 | M | 53 | 28.1 | ALD, HVC, HVB | yes | aspirin, azathioprine, coamoxiclave, cotrimoxazole, enoxaparin, entecavir, lansoprazole, Morphine Sulfate, nystatin, paracetamol, piperacillin/tazobactam, prednisolone, prograf (BD tacrolimus), senna, sertraline, testogel, tramadol, (cyclizine, lidocaine, morphine sulfate, ondansetron, paptac, tramadol as required) |
| M5 | M | 56 | 27.2 | ALD, HCV, HCC | gave up 6 weeks before breath sample | None |
| M6 | M | 53 | 33.1 | CD | no | rifaximin,ursodeoxycholic acid, citalopram, Lactulose, carvedilol, spironolactone, buscopan, enlive plus, omeprazole, micolax enema, peppermint oil |
| M7 | M | 36 | 35.0 | CD | no | furosemide, amiloride, carvedilol |
| **Study ID** | **Sex** | **Age** | **BMI** | **diagnosis** | **Smoker** | **Medications** |
| M8 | M | 67 | 25.7 | ALD | no | beclomethasone, vitamins A and D, lactulose, enoxaparin, hydrocortisone, omeprazole, paracetamol, fresnius renalyte acid concentrate, fresnius BiB ag, calogen extra, Build Up (sweet), ciprofloxacin, rifaximin, |
| M9 | M | 55 | 24.0 | PSC | no | paracetamol, codeine phosphate |
| M10 | M | 53 | 42.3 | ALD | no | lenograstim, cotrimoxazole, neomycin, vitamin B, thiamine, propanalol, lansoprazole |
| M11 | M | 67 | 24.5 | ALD | no | omeprazole, folic acid, furosamide, spironalactone, tamazepam, carvedilol |
| M12 | M | 50 | 24.1 | PSC | no | vitamin K, calcium, vitamin D |
| M13 | M | 42 | 21.8 | PSC | no | omeprazole, septrin (sulfamethoxazole), creon, questran (Cholestyramine) |
| M14 | M | 64 | 35.9 | ALD | no | propanalol, spironalactone, vitamin D (rocaltrol), omeprazole, erythromycin, |
| M15 | M | 54 | 29.8 | ALD | no | Fortimel, rifaximin, lactulose, thiamine, build up, vitamin B, lansoprazole, phosphate enema, piperacillin/tazobactan, ciprofloxacin, trimethoprim |
| M16 | M | 45 | 31.6 | ALD | yes | none |
| M17* | M | 65 | 31.2 | NAFLD, HCC | no | paracetamol |
| M18 | M | 69 | 37.0 | ALD, HCC | no | omeprazole, propranolol, thiamine, fluoxetine, vitamin B |
| M19 | M | 57 | 27.8 | HBV, HCV, HCC | gave up 2 months before breath sample | metformin, omeprazole, ramipril, amitriptyline, naproxen, viagra, bendrofluazide |
| M20 | M | 66 | 29.4 | ALD, HCC | yes | furosemide, spironolactone, lactulose, vitamin B, thiamine |
| M21 | M | 50 | 27.2 | HBV, HCC | yes | tenofovir |
| M22 | M | 71 | 24.5 | CD, HCC | no | furosemide, amiloride |
| M23 | M | 55 | 28.2 | HCV, HCC | yes | metformin, insulin, paraxatone |
|  |  |  |  |  |  |  |
| *Only patient M17 reported that they still drank alcohol | | | | | | |
