# Supplementary material for: Volatile Biomarkers in Breath Associated With Liver Cirrhosis — Comparisons of Pre- and Post-liver Transplant Breath Samples
Source: eBioMedicine. 2015 Jul 26;2(9):1243–50. doi: 10.1016/j.ebiom.2015.07.027 (PMC4588000; doi:10.1016/j.ebiom.2015.07.027)
Supplement: Supplementary Table 2 — Normalised counts per second (using area under the peak) (NCPS) for the 40 measured ion peaks which were included in the statistical analysis for patients, controls and room air. Median (lower quartile LQ, upper quartile UQ) and range (minimum–maximum) are provided. NCPS refers to normalising to 50 million counts per second for the sum of the reagent ions m/z 19 (hydronium) and m/z 37 (the protonated water dimer). Note the number of room air samples are less than those for patients and controls because some patients were recruited in the same clinic at the same time. [file mmc2.docx]

**Supplementary Table 2.** Normalised counts per second (using area under the peak) (NCPS) for the 40 measured ion peaks which were included in the statistical analysis for patients, controls and room air. Median (lower quartile LQ, upper quartile UQ) and range (minimum – maximum) are provided. NCPS refers to normalising to 50 million counts per second for the sum of the reagent ions *m/z* 19 (hydronium) and *m/z* 37 (the protonated water dimer). Note the number of room air samples are less than those for patients and controls because some patients were recruited in the same clinic at the same time.

|  | Patients (N = 31) | | Controls (N = 31) | | Room air (N = 21) | |
| --- | --- | --- | --- | --- | --- | --- |
| *m/z* | Median (LQ, UQ) | Range | Median (LQ, UQ) | Range | Median (LQ, UQ) | Range |
| *m/z* 33 | 77200 ( 64800 / 144700) | 36600 - 334700 | 56900 ( 45100 / 68700) | 32600 - 92200 | 10300 ( 8800 / 14600) | 5800 - 22300 |
| *m/z* 42 | 4300 ( 3950 / 6290) | 3590 - 78060 | 4780 ( 3830 / 37440) | 2810 - 98400 | 3030 ( 2800 / 5220) | 1550 - 10050 |
| *m/z* 43 | 86200 ( 75100 / 91200) | 46700 - 186300 | 86100 ( 63700 / 98300) | 45100 - 330700 | 29700 ( 21300 / 34400) | 10000 - 236400 |
| *m/z* 45 | 66400 ( 50000 / 84800) | 21500 - 198200 | 65700 ( 39000 / 103100) | 24400 - 281700 | 30800 ( 19800 / 40800) | 8900 - 257700 |
| *m/z* 47 | 34500 ( 31900 / 40400) | 15200 - 64100 | 34000 ( 26800 / 37000) | 22800 - 119800 | 16800 ( 12200 / 18700) | 8300 - 29000 |
| *m/z* 49 | 980 ( 720 / 1160) | 320 - 3920 | 880 ( 720 / 1160) | 310 - 1620 | 450 ( 220 / 630) | 80 - 1310 |
| *m/z* 51 | 3870 ( 2830 / 5890) | 720 - 12430 | 2480 ( 1850 / 3150) | 1270 - 6000 | 380 ( 160 / 550) | 40 - 760 |
| *m/z* 54 | 860 ( 580 / 1180) | 290 - 2390 | 1050 ( 610 / 1570) | 240 - 30380 | 260 ( 140 / 410) | 40 - 610 |
| *m/z* 55 | 85600 ( 71100 / 120400) | 37200 - 208900 | 72900 ( 58200 / 117500) | 39100 - 1114900 | 5900 ( 4500 / 7300) | 2100 - 81300 |
| *m/z* 57 | 12220 ( 10150 / 14690) | 5300 - 20300 | 10530 ( 9670 / 12220) | 6720 - 25720 | 4480 ( 3010 / 4960) | 1970 - 9750 |
| *m/z* 58 | 2750 ( 2060 / 5090) | 990 - 11320 | 2710 ( 1890 / 4590) | 1030 - 92120 | 650 ( 480 / 1130) | 270 - 8640 |
| *m/z* 59 | 359400 ( 268100 / 719700) | 158200 - 1119800 | 269600 ( 219700 / 449200) | 124600 - 1078500 | 31200 ( 20000 / 119000) | 9200 - 1616500 |
| *m/z* 61 | 50400 ( 42100 / 57300) | 13600 - 138200 | 49500 ( 36800 / 61100) | 26200 - 121200 | 13000 ( 9500 / 15700) | 5200 - 23600 |
| *m/z* 63 | 8580 ( 7370 / 10380) | 3890 - 11780 | 7650 ( 5760 / 9320) | 4760 - 13500 | 660 ( 510 / 1050) | 220 - 1640 |
| *m/z* 65 | 2340 ( 1960 / 2930) | 820 - 4110 | 1930 ( 1660 / 2390) | 940 - 29660 | 370 ( 230 / 520) | 50 - 950 |
| *m/z* 67 | 740 ( 590 / 940) | 280 - 3330 | 660 ( 490 / 850) | 280 - 1940 | 180 ( 110 / 360) | 70 - 510 |
| *m/z* 68 | 390 ( 320 / 470) | 150 - 2160 | 430 ( 370 / 490) | 130 - 4630 | 140 ( 60 / 270) | 20 - 630 |
| *m/z* 69 | 18250 ( 5800 / 25890) | 2640 - 73030 | 11420 ( 7580 / 19410) | 1890 - 43170 | 830 ( 550 / 970) | 370 - 2800 |
| *m/z* 71 | 8410 ( 5530 / 11200) | 2580 - 16230 | 6020 ( 5040 / 8130) | 2230 - 13980 | 780 ( 660 / 1090) | 340 - 2460 |
| *m/z* 72 | 720 ( 580 / 920) | 430 - 1500 | 740 ( 460 / 910) | 360 - 5940 | 230 ( 190 / 400) | 40 - 890 |
| *m/z* 73 | 11900 ( 9600 / 17300) | 7500 - 30800 | 8900 ( 7300 / 12500) | 5800 - 25600 | 3300 ( 2500 / 5000) | 1900 - 16800 |
| *m/z* 74 | 870 ( 780 / 1020) | 630 - 1800 | 780 ( 610 / 980) | 450 - 2020 | 370 ( 270 / 540) | 120 - 980 |
|  | Patients (N = 31) | | Controls (N = 31) | | Room air (N = 21) | |
| *m/z* | Median (LQ, UQ) | Range | Median (LQ, UQ) | Range | Median (LQ, UQ) | Range |
| *m/z* 75 | 4450 ( 3370 / 6830) | 1490 - 13400 | 3060 ( 2460 / 5240) | 1630 - 15240 | 1210 ( 890 / 1650) | 390 - 4040 |
| *m/z* 77 | 2590 ( 1840 / 3790) | 1010 - 7470 | 1740 ( 1330 / 2920) | 830 - 8660 | 360 ( 230 / 510) | 150 - 2260 |
| *m/z* 79 | 1550 ( 1240 / 1990) | 420 - 3150 | 1300 ( 980 / 2240) | 600 - 13060 | 410 ( 300 / 560) | 150 - 830 |
| *m/z* 81 | 5000 ( 1700 / 14170) | 800 - 61880 | 980 ( 830 / 1270) | 540 - 2270 | 330 ( 260 / 450) | 140 - 1880 |
| *m/z* 83 | 1303 ( 1019 / 1905) | 530 - 3084 | 949 ( 787 / 1556) | 520 - 2959 | 479 ( 426 / 571) | 224 - 1084 |
| *m/z* 85 | 2110 ( 1070 / 3960) | 650 - 5070 | 1800 ( 1140 / 2710) | 610 - 6810 | 400 ( 360 / 620) | 230 - 1380 |
| *m/z* 87 | 7630 ( 5780 / 11400) | 2650 - 33000 | 4030 ( 3470 / 4620) | 2190 - 15410 | 830 ( 770 / 1360) | 540 - 1630 |
| *m/z* 89 | 1480 ( 980 / 3370) | 540 - 10170 | 870 ( 690 / 1330) | 570 - 12270 | 480 ( 460 / 630) | 240 - 2750 |
| *m/z* 93 | 1890 ( 1320 / 3450) | 760 - 8300 | 1090 ( 890 / 2050) | 660 - 6350 | 570 ( 400 / 760) | 180 - 990 |
| *m/z* 95 | 1810 ( 1200 / 3070) | 770 - 10950 | 1110 ( 860 / 1580) | 630 - 3730 | 450 ( 350 / 500) | 280 - 1400 |
| *m/z* 97 | 630 ( 550 / 930) | 300 - 2010 | 570 ( 470 / 790) | 310 - 1760 | 330 ( 250 / 410) | 110 - 730 |
| *m/z* 99 | 870 ( 750 / 1250) | 450 - 5640 | 790 ( 630 / 980) | 390 - 1550 | 440 ( 360 / 630) | 80 - 820 |
| *m/z* 101 | 2940 ( 2010 / 4420) | 1180 - 6370 | 2140 ( 1820 / 3170) | 1380 - 8750 | 1140 ( 700 / 1640) | 500 - 2510 |
| *m/z* 103 | 700 ( 620 / 880) | 450 - 2690 | 690 ( 530 / 910) | 300 - 3790 | 340 ( 230 / 430) | 130 - 680 |
| *m/z* 109 | 610 ( 470 / 860) | 190 - 4920 | 560 ( 480 / 750) | 230 - 4570 | 340 ( 240 / 530) | 80 - 1690 |
| *m/z* 115 | 610 ( 520 / 860) | 410 - 2430 | 530 ( 460 / 650) | 290 - 1520 | 400 ( 230 / 450) | 120 - 630 |
| *m/z* 135 | 550 ( 490 / 1100) | 270 - 2300 | 450 ( 310 / 570) | 170 - 3750 | 330 ( 240 / 450) | 20 - 1620 |
| *m/z* 137 | 3027 ( 830 / 9286) | 278 - 40636 | 409 ( 333 / 506) | 97 - 1309 | 243 ( 72 / 387) | 43 - 855 |
