# Supplementary material for: Volatile Biomarkers in Breath Associated With Liver Cirrhosis — Comparisons of Pre- and Post-liver Transplant Breath Samples
Source: eBioMedicine. 2015 Jul 26;2(9):1243–50. doi: 10.1016/j.ebiom.2015.07.027 (PMC4588000; doi:10.1016/j.ebiom.2015.07.027)
Supplement: Supplementary Table 3 — Correlations between volatiles for the 31 patients and 30 controls in the putative marker set. Significance scores below 0.05 are highlighted in bold. [file mmc3.docx]

**Supplementary Table 3**. Correlations between volatiles for the 31 patients and 30 controls in the putative marker set. Significance scores below 0.05 are highlighted in bold.

|  |  | Patients (N = 31) | | Controls (N = 30) | |
| --- | --- | --- | --- | --- | --- |
| Volatiles/*m/z* | | Kendall's tau-b statistic | Significance | Kendall's tau-b statistic | Significance |
| Limonene | Methanol | 0.127 | 0.158 | 0.057 | 0.656 |
| Limonene | 2_pentanone | 0.144 | 0.127 | 0.166 | 0.148 |
| **Limonene** | **2-butanone** | **0.333** | **0.004** | 0.147 | 0.253 |
| **Limonene** | **Carbon disulfide** | **0.230** | **0.034** | 0.078 | 0.544 |
| **Limonene** | ***m*/*z* 89** | **0.415** | **0.001** | 0.117 | 0.363 |
| **Limonene** | ***m*/*z* 135** | **0.492** | **0.000** | **0.310** | **0.016** |
| **Methanol** | **2-pentanone** | **0.288** | **0.012** | -0.140 | 0.276 |
| Methanol | 2-butanone | 0.062 | 0.311 | -0.101 | 0.432 |
| Methanol | Carbon disulfide | 0.200 | 0.057 | 0.023 | 0.858 |
| Methanol | m89 | 0.144 | 0.127 | 0.002 | 0.986 |
| Methanol | m135 | 0.118 | 0.175 | 0.122 | 0.344 |
| 2_pentanone | 2-butanone | 0.127 | 0.158 | 0.087 | 0.498 |
| 2_pentanone | Carbon disulfide | 0.028 | 0.413 | -0.037 | 0.775 |
| **2_pentanone** | ***m*/*z* 89** | **0.290** | **0.011** | 0.067 | 0.605 |
| 2_pentanone | ***m*/*z*** 135 | 0.127 | 0.158 | 0.168 | 0.193 |
| **2-butanone** | **Carbon disulfide** | **0.544** | **0.000** | **0.452** | **0.000** |
| 2-butanone | ***m*/*z*** 89 | 0.058 | 0.323 | 0.032 | 0.803 |
| 2-butanone | ***m*/*z*** 135 | 0.153 | 0.114 | 0.110 | 0.392 |
| Carbon disulfide | ***m*/*z*** 89 | 0.006 | 0.480 | -0.074 | 0.568 |
| Carbon disulfide | ***m*/*z*** 135 | 0.067 | 0.299 | -0.032 | 0.803 |
| ***m*/*z* 89** | ***m*/*z* 135** | **0.320** | **0.006** | 0.182 | 0.159 |
